# Clinicopathological Findings of Retinoblastoma: A 10-Year Experience from a Tertiary Hospital in Kampala, Uganda

**DOI:** 10.1155/2019/5829284

**Published:** 2019-06-16

**Authors:** James Joseph Yahaya, Belson Rugwizangoga, Alex Mremi, Asafu Munema

**Affiliations:** ^1^Department of Pathology, Makerere College of Health Sciences, P.O. Box 7072, Kampala, Uganda; ^2^The University of Rwanda, P.O. Box 4285, Kigali, Rwanda; ^3^Kilimanjaro Christian Medical Center (KCMC), P.O. Box 3010, Kilimanjaro, Tanzania; ^4^Ocean Road Cancer Institute (ORCI), P.O. BOX 3592, Dar-es-salaam, Tanzania

## Abstract

**Background:**

Retinoblastoma (RB) is one of the most common cancers occurring in young children in sub-Saharan Africa. The incidence rate reported in the literature is 9,000 new cases per year, which corresponds to 1 in 15,000 births. This study aimed at analyzing the clinicopathological findings in children with RB in Uganda.

**Purpose:**

The purpose of this study was to describe and analyze the clinicopathological findings in the patients with RB.

**Design:**

This was a cross-sectional analytical study involving 234 eyeball surgical specimens from 214 patients with RB diagnosed between January 2006 and December 2015.

**Results:**

The mean age of the patients was 27.8 months (SD = 21.413, range: 1–132 months). More than half of the cases, 50.9%, presented with leucokoria. Invasion of choroid, sclera, anterior chamber, and optic nerve was found in 26.5% (*n* = 58), 51.2% (*n* = 88), 26.2% (*n* = 45), and 29.2% (*n* = 49), respectively. Twenty-six percent (*n* = 56) of the cases with intraocular tumour were at stage I and all patients with metastasis 4.7% (*n* = 11) had stage IV. The correlation between postlaminar optic nerve invasion and massive choroidal invasion was statistically significant (*P* = 0.002). Also, there was a statistical significance difference between metastasis and postlaminar invasion (*P* = 0.004).

**Conclusion:**

The majority of children with RB in Uganda present clinically with leucokoria, and their parents or guardians seek medical intervention at a later stage. Moreover, there was a noticeably significant lag period for the patients to begin treatment after the diagnosis was done.

## 1. Introduction

Retinoblastoma (RB) is the most common intraocular malignant tumour in children. RB has been found to contribute a significant proportion among the childhood malignancies especially in the developing countries. It represents approximately 4.0% of all pediatric malignancies [1]. The incidence decreases with age, most cases are diagnosed before 4 years of age, and boys suffer slightly more often than girls [2]. The incidence of RB in the USA has been reported to involve 12.14 cases per million [3]. Incidence estimates from Africa include 16 cases per million children under 5 years in Malawi [4] and 9.3 per million (age standardized) in Conakry, Guinea [5], which shows that the incidence of RB in developing countries is still higher compared to the developed countries. A recent study from Zambia has shown that the incidence of RB is 12.5% and it has been increasing among the children due to human immune virus (HIV) infection [6]. The general histopathological feature of RB is the presence of monomorphic small, roundish blue cells which may or may not form rosettes depending on the degree of differentiation [7]. Other histopathological findings commonly seen include necrosis, calcification, and Azzopardi phenomenon (surrounding the blood vessels by tumour cells) [6, 7].

The current study aimed at describing the clinicopathological findings of RB, and also it determined the correlation between conventional pathological prognostic factors and other clinicopathological findings among the patients with RB from a tertiary hospital in the central part of Uganda.

## 2. Materials and Methods

This was a cross-sectional analytical study which involved a total of 234 eyeball surgical specimens from 214 patients with histological diagnosis of RB at Mulago National Referral Hospital (MNH) in Kampala, Uganda, between 2006 and 2015. The formalin-fixed embedded paraffin (FFEP) tissue blocks were retrieved and retrospectively studied at Makerere pathology laboratory. Fourteen (5.6%) cases were excluded from the study either because of missing tissue blocks or being spoiled by insects. For every eyeball specimen included for histological evaluation, two serial sections were made at a thickness of 3 microns, and each section was stained using standard Harris' haematoxylin and eosin (H&E) stains. For pathological staging, TNM version 8 of the American Joint Committee on Cancer (AJCC) of 2017 was applied (Table 1).

Data generated were collected using a structured data collection form, and statistical analysis was done using SPSS 16.0 version (SAS Institute, Cary, NC 2010) software. Statistical association between continuous and categorical variables was computed using Fisher's exact test and Pearson chi-square (*χ*^2^) test, respectively. *P* < 0.05 was considered statistically significant.

## 3. Results

**Figure 1:**
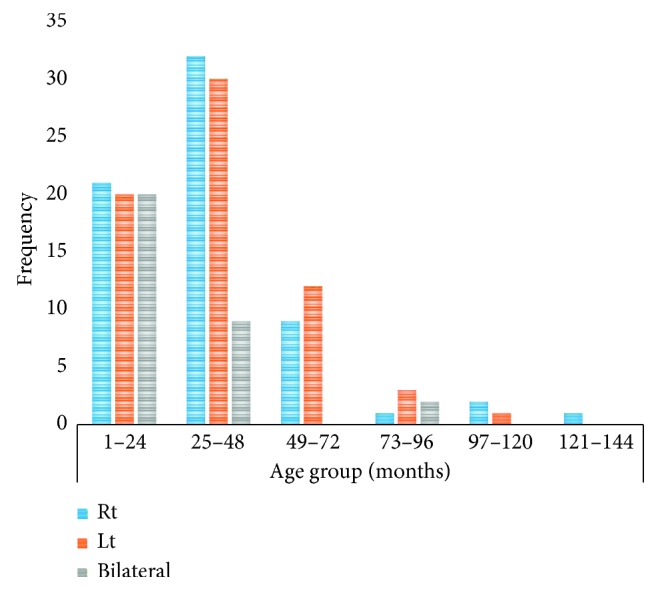
Age at presentation and laterality in retinoblastoma patients.

Overall, males and females were 57.5% (*n* = 123) and 42.5% (*n* = 91), respectively. Unilaterals comprised 90.7% with a similar ratio. The tumour involved left eye in 66.5% and the right eye in 33.5% of the cases. On the other hand, the bilaterals were 9.3% with proportion of males and females of 60% (*n* = 12) and 40% (*n* = 8), respectively (Figure 1). Positive family history for bilateral RB was documented in 9.7% (*n* = 3) cases who were all females with no known screening information for RB protein gene mutation. The mean age for our cases was 46.7 months (SD = 32.4 months, range 1–132 months). The mean age for cases with bilateral and unilateral RB was 21.4 months (SD = 19.6, range 1–96 months) and 35.4 months (SD = 24.4, range 3–132 months), respectively.

The duration from onset of symptom or sign to the time of seeking diagnosis and medical intervention ranged from 3 to 8 weeks for bilateral cases and 3 to 36 months for unilaterals, whereas the duration since diagnosis to the time of starting treatment (lag time) ranged from 3 to 12 weeks and 3 to 36 months for bilateral and unilateral cases, respectively. The most common clinical feature was leucokoria which comprised 50.9% (*n* = 109) of the patients. Proptosis was the second presenting clinical feature which comprised 31.8% (*n* = 69) of the cases (Table 2).

**Figure 2:**
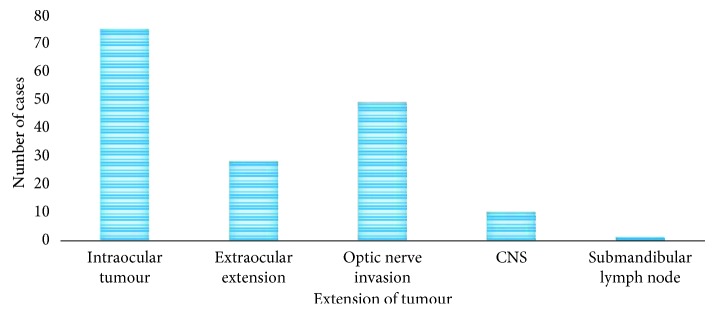
Distribution of the patients by extension of retinoblastoma tumour.

Information in the laboratory request forms and patients' files showed that during examination by computer tomography (CT) scan, MRI, and bone marrow aspiration, 66.7% (*n* = 156) of the patients had intraocular tumour and 28.6% (*n* = 67) of them had extraocular tumour. Those with extraocular involvement, 5.6% (*n* = 13), 5.3% (*n* = 12), 11.5% (*n* = 27), and 6.4% (*n* = 15), had orbital bone extension, positive transected head of the optic nerve, and extraocular muscle involvement, respectively. Metastatic disease was found in 4.7% (*n* = 11) patients involving central nervous system (CNS) and submandibular lymph node infiltration (Figure 2). CNS involvement was frequently diagnosed by performing lumber puncture for CSF and then testing by means of a cytospin machine together with brain MRI when it was possible. Enucleations were performed in 83.3% (*n* = 195) of the patients and 18.2% (*n* = 39) had exenteration.

Fundoscopic examination reports recorded in the requisition laboratory forms and patients' files indicated that 42.1% (*n* = 90) had exophytic growth pattern, 23.4% (*n* = 50) had endophytic growth pattern, and the remaining 10.7% (*n* = 23) cases had diffuse growth pattern.

**Figure 3:**
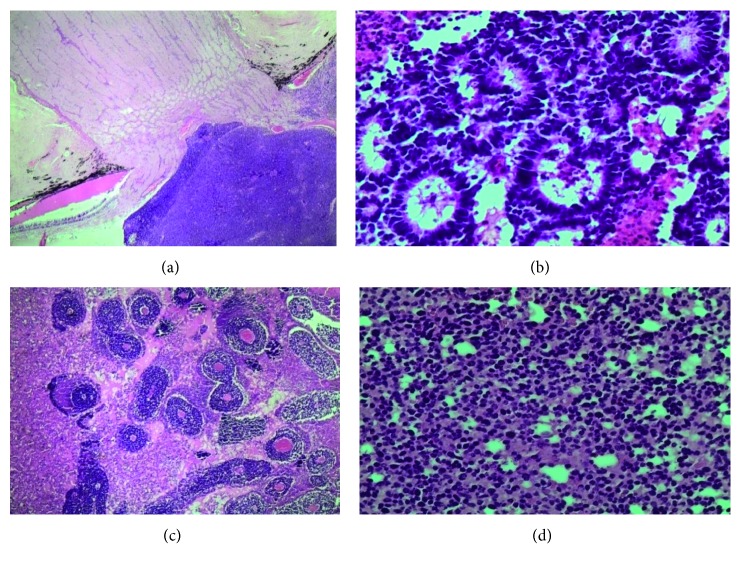
(a) Lamina cribrosa optic nerve invasion. (b) Tumour with Flexner–Wintersteiner rosettes. (c) Tumour with pseudorosettes. (d) Poorly differentiated.

Table 3 and Figure 3 represent the histological findings and the invasion of the tumour to different ocular parts. Almost half of the eyeball specimens, 47.9% (*n* = 112), showed poorly differentiated tumour and majority of them had pseudorosettes, 54.3% (*n* = 127). Other histological features were as shown in Table 3.

Local invasion among the cases was present in 42.7% (*n* = 100) and the remaining, 23.9% (*n* = 56), did not show local invasion. Choroidal invasion was found in 27.3% (*n* = 47) and it was categorized into two groups: focal and massive choroidal invasion. Focal choroidal invasion was present in 11.0% (*n* = 19) of the eyeballs, and it is considered when the tumour focus is <3 mm in diameter without involving the sclera. Massive choroidal invasion was considered when tumour focus was ≥3 mm in diameter with a possibility of involving the sclera which was found in 22.7% (*n* = 28) eyes. Scleral invasion was found in 22.7% (*n* = 39). Of the cases with scleral invasion, 18% and 4.7% had intrascleral and extrascleral invasion, respectively. Anterior chamber involvement was found in 21.5% (*n* = 37) comprising of 7.0% and 14.5% for iris and ciliary body, respectively.

Optic nerve invasion was present in 28.5% (*n* = 49). Of which, 12.8% had prelamina cribrosa (Figure 3(a)), 10.4% had postlamina cribrosa involvement, and the remaining 5.3% had involvement of the surgical margin.

**Figure 4:**
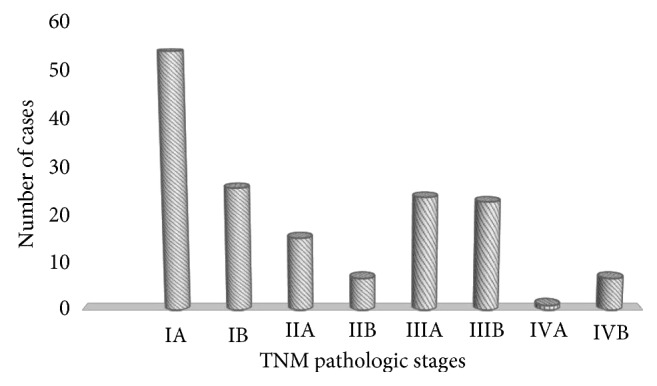
Pathologic TNM staging of the patients with retinoblastoma tumour.

Figure 4 represents the different pathologic stages of the patients in this study. The majority of the patients had pathologic stage ranging from I to III which comprised 66.7% (*n* = 156). Stages I, II, and III were present in 23.9% (*n* = 56), 23.5% (*n* = 55), and 19.2% (*n* = 45) of the patients, respectively. Stage IV disease was found in 33.3% (*n* = 78) patients. Most of stage IV patients, 28.6% (*n* = 67), had extraocular disease and the remaining, 4.7% (*n* = 11), cases had distant spreading of the disease.

Correlation of variables studied in this study is shown in Table 4. All the cases with metastasis (*n* = 11), were poorly differentiated and only 2.3% (*n* = 5) of them were moderately differentiated and the difference was statistically significant (*P*=0.002). The eyeball specimens with necrosis rate >50% having metastasis were 29.4% (*n* = 63) compared to 9.3% (*n* = 20) specimens with necrosis rate <50% with metastasis, and the difference was statistically significant (*P*=0.002). Among the cases with optic nerve invasion, 22.4% (*n* = 11) had metastasis whereas 6.1% (*n* = 3) of cases without optic nerve invasion had metastasis and the difference was not statistically significant (*P*=0.39). When postlaminar cribrosa invasion of the optic nerve was correlated with metastasis, it was found that the correlation was statistically significant (*P*=0.004). Of the 234 eyeball specimens, 87.2% (*n* = 207) were poorly differentiated and constituted pathologic stages III and IV compared to 12.2% (*n* = 27) of the cases with differentiated tumour which had stages I and II and the difference was statistically significant (*P*=0.001). The correlation between massive choroidal invasion postlaminar optic nerve invasion was statistically significant (*P*=0.002). Likewise, when postlaminar optic nerve invasion was correlated with metastasis, the association between the two variables was also statistically significant (*P*=0.004).

## 4. Discussion

The male-to-female ratio of 1.35 : 1 for this study was similar to the studies done in Nepal (1.5 : 1) and Uzbekistan (1.3 : 1) [9, 10] while in other series, authors have reported slight male predominance [5, 11]. This variation may be due to bias in referral situations as well as environmental variations across the regions [12]. The low proportion of patients with bilateral RB in this study and high proportion for unilateral cases in this study are in keeping with several findings reported from different settings in literature such as Kenya (37%), India (43%), and Malawi (36%) [13–15].

The age range of the cases in this series was almost close to those reported in other studies. Most of the studies have found that the age of most of the patients with RB range from 1 to 8 years compared to the age range of our patients which ranged from 1 month to 11 years [13, 15]. However, one study has shown a RB patient of 23 years [16] which is also more than the age of the oldest patient in this study which was 11 years. The mean age at diagnosis for bilateral RB in this study was slightly lower than that for unilateral RB which is similar to what has been reported by Stannard and coworkers in South Africa [17]. Similar findings have also been reported in other studies regarding the occurrence of bilateral RB compared to unilateral ones. The reason for early development of bilateral RB is that, regarding Knudson's second hit theory for deletion of the RB gene, one deletion is required for the development of the disease. Unilateral RB requires two deletions, and therefore, the disease develops later [18]. The most common presenting clinical feature was leucokoria similar to many findings that have been reported in DRC (67.5%), Kenya (71%), and Ghana (87%) [11, 13, 19]. For instance, Gichigo et al. reported leucokoria of 71% in RB patients [13]. This finding was also similar to the finding in the study done by Kazadi et al. [11] in Democratic Republic of Congo (DRC) where leucokoria was found in 67.5% of patients with RB. In the study done by Essuman and associates in Ghana, it was found that leucokoria was present in 87% of the cases with RB which is much higher than what was found in this study [19]. Leucokoria has also been reported to be the common clinical feature among patients with RB even in developed countries. For example, in the study done by Eagle [20] in the USA, he found that 66.8% of the cases with RB were presenting with leucokoria clinically. Therefore, fundoscopy is best done after delivery in order to detect early tumours. By the time there is leucokoria, tumour usually fills the eye, but looking for leucokoria in any children's clinic is important, which can be done by any health professional and can detect tumours before they spread any further.

In this study, proptosis was the second most common clinical feature comprising 31.8% which is higher than 20% which was reported by Gichigo et al. in Kenya [13]. A much higher proportion of RB patients have been reported in DRC by Kazadi et al. [11]. In their study, they found that 55% of the patients already had proptosis at initial diagnosis. Among the reasons already known for this high proportion of patients presenting with proptosis in developing countries include delay of the patients to seek medical services from the first day of seeing some of the clinical features, belief in traditional medicine, lack of education, poor health seeking behavior, lack of paediatricians and ophthalmologists at lower health facilities, and low socioeconomic status (SES) [21].

It has been reported that strabismus is more frequent in younger patients with macular involvement [13, 17]. We also found that, among the patients with strabismus in this study, most of them had macular involvement. Chong et al. [22] reported that, in the Republic of Korea, the proportion of patients with strabismus was 17.5% compared to that of our patients of whom strabismus was present in 19.6%.

The proportion of cases with rosette formation (45.8%) in this study is higher than 17.4% that was found by Owoeye et al. [23] in Ilorin but lower than 52% that was reported by Reddy and Anusya [24] among patients with RB in Malaysia.

Differentiation of RB in our patients was as poor as that which has been observed in other settings especially those in developing countries. Nyaywa and associates [6] in Zambia reported only 6.7% of the cases with RB who were well differentiated which is lower than 19.7% of the cases with well-differentiated cases which was found in the current study. Owoeye et al. [23] reported 17.4% of well-differentiated cases which is almost similar to what was found in the present study. The finding of poorly differentiated cases in this study was 47.9% which was much lower than 97% and 82.6% which were reported in the series of Mukhtar et al. [25] and Owoeye et l [23], respectively, but similar to 50% and 58% reported by Gupta et al. [26] and Yeole and Advani [27], respectively. Studies have shown that a large proportion of patients with RB in developed countries are differentiated. For example, Eagle and associates [20] in the USA reported 41% of patients with differentiated RB. This noticeable difference of tumour differentiation in RB between developing and developed countries has been associated with better health seeking behavior, availability of paediatricians and ophthalmologists at lower health facilities, and reasonable socioeconomic status (SES) in developed countries all resulting in diagnosis and treatment [21].

All these factors contribute at large for early detection of the tumour as compared to most of the settings in the developing countries. Differentiation in RB seems to have no prognostic role. Studies have shown that the degree of differentiation is not associated with the adverse prognostic factors such as stage, optic nerve invasion, massive choroidal invasion, and metastasis. For example, Zoroquiain and associates [28] reported no association between the degree of differentiation and metastasis among the patients and also differentiation did not predict the prognosis of the patients independently. There is no known reason why the degree of differentiation does not correlate with many other prognostic factors.

Necrosis was correlating with the degree of differentiation (*P*=0.002), where the majority of the cases that were poorly differentiated had necrosis rate >50%. This finding is in line with what was found by Kashyap et al. [29] in India where they found that the association between extensive necrosis and tumour differentiation was highly statistically significant (*P*=0.001). This is due to the fact that RB tends to be poorly vascularized as it advances in grade, thus presenting with extensive necrosis. In a study done by Kerimogglu et al. [30], it was found that the correlation between differentiation and extensive necrosis was statistically significant (*P*=0.044) which is also in keeping with what we found in this study. It was also found that the degree of extensive necrosis was related to metastasis.

Choroidal invasion, especially massive one, increases the chances of metastasizing of RB from 16% to 36% when the invasion is concomitant with optic nerve invasion. The proportion of cases with choroidal invasion of 27.3% in this series was greater than 16.9% and 18% which were reported by Mukhtar and Kagame [25] in Mbarara, Uganda, and Reddy and Anusya [24] in Malaysia, respectively. The difference could be due to the difference in samples between the two series. The extent of choroidal invasion in our series was less than 40% which was reported by Reddy and Anusya [24]. When choroidal invasion was correlated with optic nerve invasion, it was found that massive choroidal invasion was associated with postlaminar cribrosa invasion of the optic nerve (*P*=0.002). This finding is in keeping with what was reported by Brennan et al. [31] in which the association between the two variables was highly statistically significant (*P* < 0.001).

Massive choroidal invasion, unlike focal one, has been reported as the high risk factor for RB and it can predict the clinical outcome of patients. Studies have shown that choroidal invasion can predict presence of metastasis more reliably than optic nerve invasion. In the series of Darwich and associates [32], it was reported that patients with choroidal invasion were more likely to develop systemic metastasis compared to the ones with or without optic nerve invasion (*P*=0.0001). Choroidal invasion has also been found to correlate with molecular prognostic markers such as TP53 unlike other high risk factors (HRF) as it was once reported by Seema and associates [33] in India.

Postlaminar optic nerve invasion has been found to be one of the HRF in patients with RB disease with the highest prediction ability for metastasis. A slightly low optic nerve invasion of 21.7% was reported in Ghana compared to 28.5% in the current study [19]. Much higher cases with optic nerve invasion of 33.2% and 69.7% have been reported by Mukhtar and coworkers [25] in Uganda and Owoeye et al. [23] in Nigeria, respectively. Optic nerve invasion appears to predict metastasis and mortality rate especially when the extent of invasion is considered [34]. Optic nerve involvement is found in 25%–45%; however, its risk on outcome appears to be limited to the involvement beyond the lamina cribrosa and to the optic nerve surgical margin [34]. When the extent of optic nerve invasion was not considered and association with metastasis was done in this study, it was found that there was no association with metastasis (*P* = 0.39) although the trend of cases with optic nerve invasion to develop metastasis was higher than the ones without optic nerve invasion. This observation was different from the study of Mendoza and coworkers [35] in which there was correlation between optic nerve invasion and metastasis (*P* = 0.0004), and when postlaminar optic nerve invasion was associated with metastasis, the association was highly statistically significant (*P* = 0.0001) similar to the observation in this study (*P* = 0.004). Optic nerve and choroidal invasion both have shown strong and independent prediction of metastasis in most of the studies.

## 5. Conclusions

Majority of patients with RB in Uganda present clinically with leucokoria and proptosis, and most of them tend to have poorly differentiated tumour. Patients with optic nerve invasion almost all have systemic metastasis especially CNS involvement. Massive choroidal invasion and postlaminar cribrosa invasion of the optic nerve are powerful predictors of metastasis in RB.

## Figures and Tables

**Table 1:** Pathologic TNM (pTNM) version 8 of the American Joint Committee on Cancer staging [8].

pT	pN	pM
pTX: Pprimary tumour cannot be assessed	pNX: Rregional lymph nodes cannot be assessed	pM1: Distant metastasis with histopathological confirmation
pTO: Nno evidence of primary tumour	pNO: Nno regional lymph node involvement	pM1a: histopathological confirmation of tumour at any distant site (bone marrow, liver, or others)
pT1: iintraocular tumour(s) without any local invasion, focal choroidal invasion, or pre- or intra-laminar invasion of the optic nerve head	pN1: regional lymph node involvement	pM1b: histopathological confirmation of tumour in the CSF fluid or CNS parenchyma
pT2: intraocular tumour(s) with minimum local invasion		
pT2a: intraocular tumour(s) with local invasion-focal choroidal invasion, pre- or intra-laminar invasion of the optic nerve head		
pT2b: intraocular tumour(s) with invasion of stroma iris and/or trabecular meshwork and/or Schlemm's canal		
pT3: intraocular tumour(s) with significant local invasion		
pT3a: iintraocular tumour(s) with massive choroidal invasion in largest diameter, or multiple foci of focal choroidal invasion totaling >3 mm, or any full thickness choroidal invasion		
pT3b: intraocular tumour(s) with post-laminar invasion of the optic nerve but not up to the end of its surgical margin		
pT3c: intraocular tumour(s) with any partial thickness involvement of sclera within the inner two-thirds		
pT3d: intraocular tumour(s) with full -thickness into the outer -third of the sclera and/or invasion into or around emissary channels		
pT4: evidence of extraocular tumour: positive surgical margin of the optic nerve, full -thickness of the sclera, extra-ocular muscle, orbital bone, conjunctive, or eyelids		

**Table 2:** Clinical features at presentation in retinoblastoma patients (*n* = 234).

Clinical feature	Number of patients (*N*)	Percentage (%)
Leucokoria	109	50.9
Proptosis	69	31.8
Strabismus	42	19.6
Tearing/discharge	12	5.6
Reddish eye(s)	7	3.2
Pain	25	11.7
Orbital cellulitis	5	2.3
Buphthalmos	11	5.1
Loss of vision	27	12.6
Photophobia	7	3.3

**Table 3:** Histological features of the patients (*n* = 234 eyes).

Histological features	Number of eyes (*N*)	Percentage (%)
Tumour differentiation		
Well differentiated	46	19.7
Moderately differentiated	76	32.5
Poorly differentiated	112	47.9

Formation of rosettes		
Flexner–Wintersteiner	36	15.4
Homer Wright	71	30.3
Pseudorosettes	127	54.3
Fleurettes	—	—

Tumour invasion		
Optic nerve	49	28.5
Choroidal	47	27.3
Scleral	39	22.7
Anterior chamber	37	21.5

Necrosis		
<50%	51	21.8
>50%	83	35.5

Dystrophic calcification		
Yes	69	29.5
No	165	70.5

Cholesterol clefts		
Yes	34	14.5
No	200	85.5

**Table 4:** Association of clinicopathological variables evaluated in the study (*n* = 234 eyes).

Association evaluated	Number of eyes (*N*)	*P* value (95% CI)
Poorly differentiated RB and metastasis	234	*P*=0.8^*∗*^(0.6 − 3.1)
Necrosis rate (>50%) and differentiation	234	*P*=0.033^*∗*^(47.6 − 62.7)
Optic nerve invasion and metastasis	234	*P*=0.39^†^(0.2 − 4.9)
Postlaminar optic nerve invasion and metastasis	234	*P*=0.004(3.45 − 8.71)
Exenteration and pT stage	234	*P*=0.001^*∗*^(33.7 − 41.4)
Massive choroidal invasion and postlaminar optic nerve invasion	234	*P*=0.002^*∗*^(0.345 − 0.891)
Massive choroidal invasion and metastasis	234	*P*=0.01(3.567 − 6.195)

^†^Fisher's exact test, ^∗^chi-square test, pT—pathologic stage, RB—retinoblastoma, and CI—confidence interval.

## Data Availability

The data used to support the findings of this study are available from the corresponding author upon request.
